# An Optimized Ultra-Low-Dose Imaging Protocol for Endovascular Aortic Repair Significantly Reduces Radiation and Contrast Exposures

**DOI:** 10.3390/jcm15103796

**Published:** 2026-05-14

**Authors:** Bharti Singh, Umar Sadat, Angelos Karelis, Björn Sonesson, Nuno V. Dias

**Affiliations:** 1 Vascular Center, Department of Thoracic and Vascular Surgery, Skåne University Hospital, Inga Marie Nilssons Gata 47, 214 28 Malmö, Sweden; umar.sadat@nhs.net (U.S.); karelisangelos@gmail.com (A.K.); b.sonesson@gmail.com (B.S.); nunovdias@gmail.com (N.V.D.); 2 Department of Clinical Sciences, Lund University, P.O. Box 50332, 202 13 Malmö, Sweden

**Keywords:** endovascular aortic repair, radiation dose, dose-area product, fusion imaging, contrast volume, ALARA principle, occupational exposure, workflow optimization

## Abstract

**Objective**: To evaluate the impact of a systematic, multi-component ultra-low-dose imaging protocol on radiation and contrast exposure during endovascular aortic repair (EVAR) across diverse anatomical complexities. **Methods**: In this retrospective cohort study, 331 consecutive EVAR procedures at a tertiary vascular center were analyzed. Patients treated with an integrated ultra-low-dose protocol (Group A, *n* = 228) incorporating 2D/3D fusion navigation, low-frame-rate fluoroscopy (3.75 frames/s), restricted digital subtraction angiography (DSA), structured collimation, and routine CO_2_ angiography were compared with historical controls treated with a standard low-dose protocol (Group B, *n* = 103) where the frame rate was the same and CO_2_ was only used for fusion registration. Primary endpoint was total dose-area product (DAP). Secondary endpoints included component DAP values, fluoroscopy time, contrast volume, and technical success. **Results**: Group A demonstrated a 71% reduction in median total DAP (57.9 vs. 199.3 Gy·cm^2^, *p* < 0.001), driven primarily by an 79% reduction in DSA-associated and 45% fluoroscopy-associated radiation. Contrast volume decreased by 20% (101 vs. 126 mL, *p* < 0.001) without increased fluoroscopy time (57 vs. 64 s, *p* = 0.278). Technical success remained comparable (86% vs. 87%, *p* = 0.809). Reductions were consistent across all repair types, most pronounced in infrarenal repairs with iliac-branch-devices (70% DAP reduction). Within Group A, a dose–response relationship was evident: procedures with ≥70% ultra-low-dose DSA utilization achieved 61% lower radiation than those with <70% adherence. **Conclusions**: A protocolized, system-level ultra-low-dose imaging workflow achieves substantial, durable reductions in radiation and contrast exposure during EVAR of varying complexity without compromising technical success. This integrated approach represents a scalable strategy for enhancing safety for patients and procedural staff alike.

## 1. Introduction

Endovascular aortic repair (EVAR) has become the standard of care for aortic aneurysm treatment, offering reduced perioperative mortality and shorter hospitalization compared to open repair [1,2,3,4]. The evolution toward fenestrated, branched, and thoracoabdominal repairs has expanded therapeutic options but increased procedural complexity and dependence on fluoroscopic guidance [5].

This reliance introduces significant iatrogenic radiation exposure, constituting a dual-risk paradigm for patients and staff [6,7]. For patients, exposure contributes to cumulative stochastic risk, including malignancy, with complex procedures correlating with higher effective doses. Concurrently, occupational exposure remains a critical concern for high-volume operators, with evidence suggesting elevated risk of DNA damage and certain malignancies compared to non-exposed healthcare workers [8,9,10]. The persistence of iodinated contrast as a contributor to nephropathy further compounds patient-specific risks, particularly in those with renal impairment [11].

Consequently, minimizing exposure aligns with the as low as reasonably achievable (ALARA) principle, although its implementation often relies on piecemeal, operator-dependent techniques rather than systematic integration [12,13].

Building upon foundational work demonstrating the feasibility of fusion imaging [14], this study introduces a comprehensive, protocolized imaging workflow designed to fundamentally alter default imaging behavior. We hypothesize that integrated system-level optimization would yield greater and more consistent reductions in both radiation and contrast exposure compared to conventional strategies, without compromising procedural efficacy.

## 2. Materials and Methods

### 2.1. Study Design and Patient Selection

This retrospective cohort study was conducted at a tertiary vascular referral center equipped with a fixed hybrid operating theater (Artis Zee, Siemens Healthcare, Erlangen, Germany). Consecutive patients undergoing elective and emergent EVAR were included from two distinct periods based on the institutional imaging protocol in use. The study was approved by the Swedish Ethical Review Authority (DNR 2014/732 Date: 9 December 2014 and 2016/35 Date: 18 January 2016). Patients were allocated to study groups according to an intention-to-treat principle, based on the institutional imaging protocol applied at the time of the procedure.

**Figure 1:**
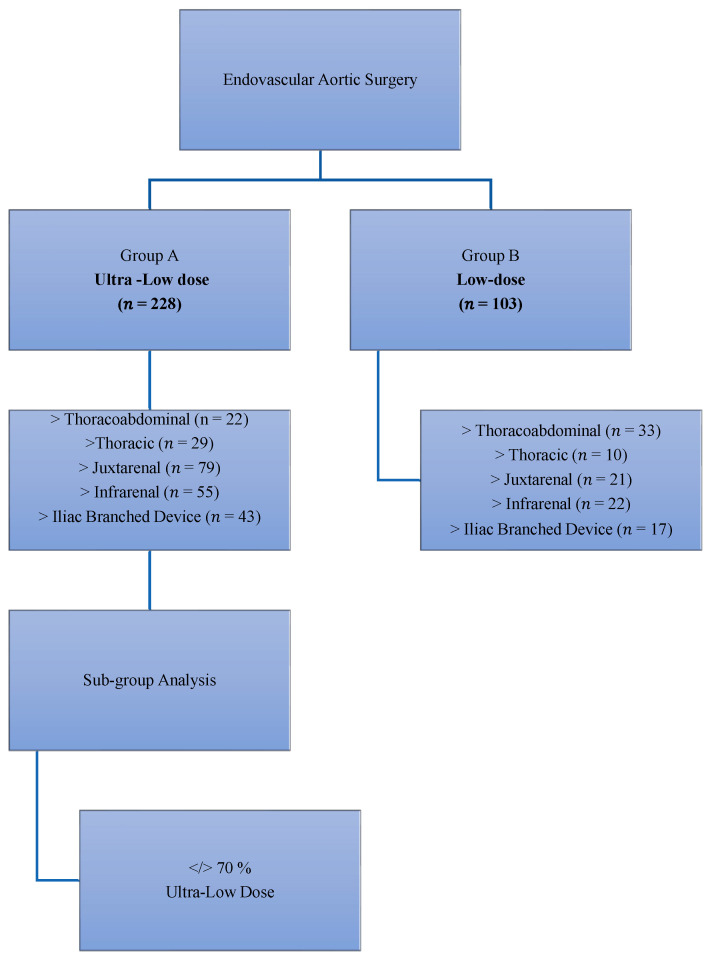
**Study design and patient allocation.** Flowchart of the study cohort undergoing endovascular aortic repair, stratified according to imaging protocol: ultra-low-dose (Group A) and low-dose (Group B). Procedural subtypes are shown for each group. A subgroup analysis within the ultra-low-dose cohort compared cases with >70% versus <70% of imaging series performed using the low-dose protocol.

Group A (ultra-low-dose protocol, *n* = 237) comprised patients treated for 3.5 years. Group B (standard low-dose protocol, *n* = 103) served as historical controls, treated for 1.5 year before and had been previously analyzed for the effects of the introduction of fusion imaging and low-frame-rate fluoroscopy [14]. Procedures performed outside the hybrid suite, those with incomplete radiation data, and aortic arch repairs (exclusive to Group A, *n* = 9) were excluded to ensure procedural comparability. Patient selection is detailed in Figure 1. All procedures in both groups were performed by the same team of experienced vascular surgeons.

### 2.2. Imaging Protocols: Technical Specifications

#### 2.2.1. Group A (Integrated Ultra-Low-Dose Protocol)

Preoperative Planning: CTA (0.6–0.75 mm slices) was reconstructed using Syngo X Workplace (Siemens Healthcare, Erlangen, Germany). Branch vessel ostia were annotated on the 3D model.

Two-Dimensional/Three-Dimensional Fusion Navigation: After arterial access, registration was performed using two single images, as close to 90 degrees apart as possible, where the skeleton was aligned with the preoperative 3D model, eliminating the need for cone-beam CT (CBCT) for registration.

Fluoroscopy: Pulsed at 3.75 frames/second with ultra-low-dose per-pulse settings developed by the manufacturer together with the investigators.

Digital subtraction angiography (DSA): Use was restricted and protocolized. Runs were acquired at 2 frames/second for the initial 6 s, followed by 1 frame/second. CO_2_ angiography via automated injector (Angiodroid^®^, Angiodroid Srl, San Lazzaro di Savena, Bologna, Italy) was employed routinely. Iodinated contrast (Omnipaque 140 mg I/mL) reserved for select imaging. Even here, ultra-low-dose per-pulse settings were used developed by the manufacturer together with the investigators.

Collimation: Active, structured collimation was mandated throughout.

Operator Discretion: Override to standard dose settings was permitted only in cases of insufficient image quality, with post-procedural review.

#### 2.2.2. Group B (Standard Low-Dose Protocol)

3D/3D Fusion: Registration required a 5 s low-dose CBCT.

Fluoroscopy: 3.75 frames/second with standard low-dose settings.

DSA: Utilized liberally with the same iodinated contrast and frame rate as above for Group A but with standard manufacturer low-dose settings (CARE); CO_2_ was not routinely used beyond the manual injection done for fusion registration adjustment as described elsewhere [14].

Other Settings: Collimation and frame rates were applied as much as possible at operator discretion.

### 2.3. Endpoints and Definitions

Primary Endpoint: Total dose-area product (DAP, Gy·cm^2^), defined as the combined fluoroscopy and DSA dose, excluding all CBCT-related exposures in both groups.

Secondary Endpoints: Fluoroscopy DAP (Gy·cm^2^); DSA DAP (Gy·cm^2^); fluoroscopy time (seconds); contrast volume (mL); iodine dose (g). Technical Success.

Technical Success: Defined according to Society for Vascular Surgery reporting standards as successful endograft deployment, complete aneurysm exclusion, absence of type I or III endoleak, and patent branch vessels as intended.

Procedural Classification: Repairs were categorized as: thoracoabdominal (TAAA), juxtarenal, infrarenal, iliac branch device with infrarenal (IBD + Infrarenal), or thoracic (TEVAR).

### 2.4. Statistical Analysis

Continuous variables are presented as median with interquartile range (IQR) and compared using the Mann–Whitney U test. Categorical variables are presented as counts (percentages) and compared using χ^2^ or Fisher’s exact test. A two-sided *p*-value < 0.05 was considered statistically significant. All analyses were performed using SPSS version 29.0 (IBM Corp., Armonk, NY, USA). Scatterplots were used to assess the relationship between adherence to the low-dose DSA preset and total procedural dose-area product (DAP) within Group A. A locally weighted scatterplot smoothing (LOESS) curve was applied to illustrate the overall trend, and boxplot overlays were used to summarize the distribution of DAP values.

## 3. Results

### 3.1. Baseline Characteristics

The cohorts were well matched for age, sex, body mass index, and baseline renal function (Table 1). Hypertension was more prevalent in Group B (78% vs. 63%, *p* = 0.005). Case mix differed between periods, reflecting evolving clinical practice: Group B had a higher proportion of thoracoabdominal repairs (32% vs. 9%), while Group A included more juxtarenal repairs (33% vs. 20%). This heterogeneity necessitated subtype analysis to isolate the protocol effect from case complexity.

### 3.2. Radiation and Contrast Exposure: Overall Cohort

**Figure 2:**
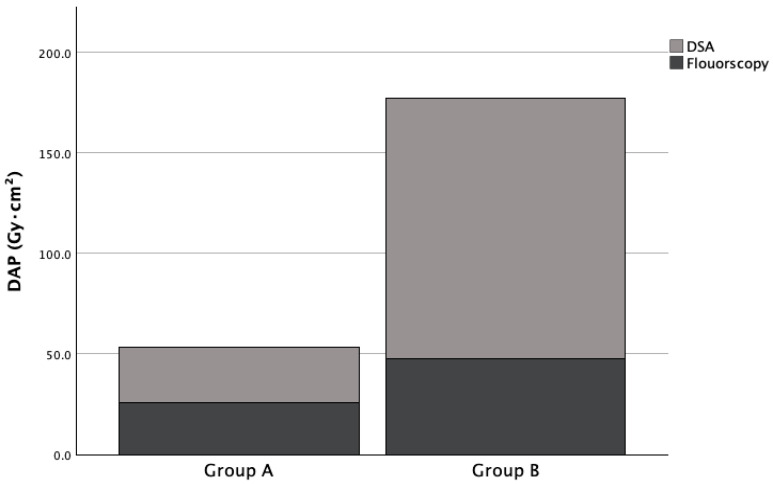
**Relative contribution of imaging components to total radiation dose.** Stacked bar chart illustrating the median dose-area product (DAP) from fluoroscopy and digital subtraction angiography (DSA) for Group A and Group B. The visualization highlights the dramatic reduction in DSA-associated radiation, which drives the overall 62% total DAP reduction.

Implementation of the ultra-low-dose protocol resulted in a 71% reduction in median total DAP (57.9 vs. 199.3 Gy·cm^2^, *p* < 0.001; Table 2). This reduction was driven predominantly by an 79% decrease in DSA-associated radiation (27.2 vs. 129.9 Gy·cm^2^, *p* < 0.001; Figure 2). Concurrently, median contrast volume was reduced by 20% (101 vs. 126 mL, *p* < 0.001) without a significant change in the iodine dose exposure. Similarly, the fluoroscopy time was similar (57 vs. 64 s, *p* = 0.278), but the fluoroscopy DAP-associated radiation exposure was reduced by 46% (26.0 vs. 47.5 Gy·cm^2^, *p* < 0.001).

### 3.3. Subgroup Analysis by Procedure Type

The protocol’s efficacy was consistent across the anatomical spectrum of aortic repairs (Table 3). The magnitude of DAP reduction ranged from 36% in thoracic repairs (*p* = 0.058) to 70% in infrarenal repairs with IBD (*p* < 0.001). Contrast volume reduction varied by subtype, with the most significant reduction observed in IBD procedures (36%, *p* = 0.005).

### 3.4. Dose–Response Relationship and Protocol Adherence

**Figure 3:**
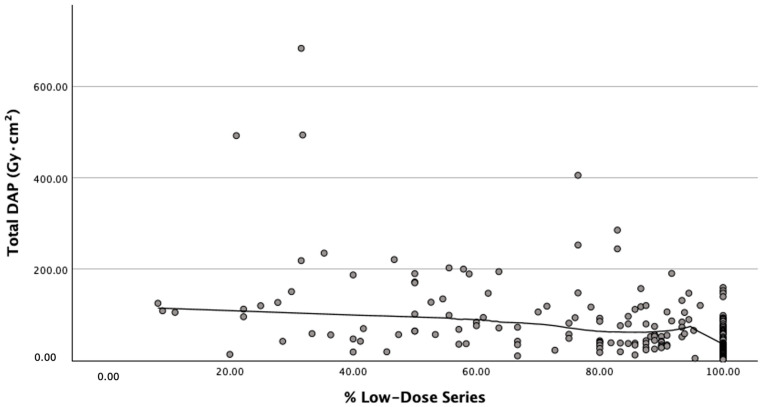
**Dose–response relationship: protocol adherence and radiation dose.** Scatterplot with boxplot overlays demonstrating the inverse relationship between adherence to the low-dose DSA preset (percentage of DSA runs using ultra-low-dose settings) and total procedure DAP within Group A. A locally weighted scatterplot smoothing (LOESS) line illustrates the trend of decreasing radiation dose with increasing protocol adherence.

Within Group A, we analyzed the impact of adherence to the ultra-low-dose DSA preset. Procedures where ≥70% of DSA runs utilized the ultra-low-dose setting (*n* = 149) achieved a 61% lower median total DAP compared to those with <70% adherence (*n* = 69) (41.4 vs. 106.1 Gy·cm^2^, *p* < 0.001), with a corresponding 25% reduction in contrast volume (91 vs. 121 mL, *p* = 0.002) (Table 4). Fluoroscopy time and technical success rates were not different between adherence groups, demonstrating that greater protocol compliance yielded greater dose reduction (illustrated in Figure 3) without adverse procedural effects. BMI did not differ in these two subgroups (26.9 vs. 27.4, *p* = 0.521).

### 3.5. Technical Success

Overall technical success was similar between Group A and Group B (86% vs. 87%, *p* = 0.809). Subtype analysis revealed no statistically significant differences in technical success for any repair category, confirming that the substantial radiation and contrast reductions did not compromise procedural efficacy (Table 3).

## 4. Discussion

This study demonstrates that a systematically implemented, multi-component ultra-low-dose protocol achieves not only a profound reduction in radiation exposure (more than two thirds) but also a significant reduction in contrast volume during EVAR, without compromising technical success across the anatomical spectrum of aortic repairs.

Radiation exposure during EVAR varies widely across studies, with complex procedures frequently associated with high DAP levels. However, as highlighted by Hertault et al., substantially lower doses can be achieved with optimized imaging techniques and adherence to dose-reduction principles. Notably, the DAP values in the present study fall within the lowest range reported, particularly for infrarenal EVAR, suggesting that our protocol achieves radiation levels at the lower boundary of what is currently attainable [15], even if the hardware was not the most modern.

*From Incremental Adjustment to System-Level Redesign.* The magnitude of radiation reduction—particularly the almost 80% decrease in DSA-associated DAP—exceeds what is typically achieved through isolated modifications such as frame-rate reduction or collimation alone [12,13]. This indicates that the benefit arises not from individual techniques, but from their integration into a default low-exposure workflow. The protocol is complementary to what is achieved by re-engineering the imaging sequence with the establishing of 2D/3D fusion registration as the default even in systems that are not primarily prepared to do it [12,14,16]. In order to focus on the effects of the X-ray settings, the CBCT doses were excluded—both the one used in the historical group for the registration of the fusion and the one done systematically for completion control. Importantly, the development of the ultra-low dose protocol underlined the need for a continuous and close collaboration with the manufacturers of the angiographic equipment who have the technical knowhow of the image optimization for the specific hardware.

*The Dose–Response Relationship: Internal Validation and Implementation Insight.* The finding within the protocol cohort that greater adherence (>70% low-dose DSA use) yields a further 61% reduction in DAP is critical. It provides strong internal validation of causality—the dose savings are directly tied to protocol utilization, not to secular trends or case selection. This relationship also offers a practical, auditable metric for centers adopting similar workflows: monitoring the proportion of low-dose DSA acquisitions can serve as a real-world measure of protocol fidelity and effectiveness.

*Clinical Safety: Renal and Occupational Implications.* Contrast volume was reduced by a fourth, which should be always strived after in EVAR procedures, particularly in patients with impaired renal function, where alternative imaging strategies aimed at minimizing contrast use have been proposed [17]. The consistent efficacy across complex repairs (juxtarenal, thoracoabdominal) confirms that contrast minimization does not come at the expense of procedural accuracy in challenging anatomy. This reduction was more pronounced when IBDs were used which is in line with the good quality of the imaging obtained in this type of repairs as previously reported [18]. Furthermore, the 71% reduction in patient DAP translates to a proportional decrease in scatter radiation exposure for the procedural team [19]. This is particularly important since there was a significant reduction of the fluoroscopy-related exposure which is the moment where the operators need to be in close proximity to the source of the scatter radiation—the patient. This as opposed to the DSA where the systematic use of power injectors allows for the staff to move away from the exposure source [19]. Although not directly measured, this is likely having a positive impact on the occupational safety—which is ethically and practically imperative for high-volume operators [7,8,19]. Moreover, further improvements may be obtained in the future if the use of radiation-free navigation tools prove their intended value [20,21,22]. Interestingly, the use of optimized CO_2_ specific acquisition programs allowing for the systematic use of this contrast agent without the increase in radiation exposure that was previously thought.

*Limitations and Interpretation.* The retrospective, single-center design limits generalizability, though the internal dose–response gradient mitigates concern over unmeasured confounding. Furthermore, the use of a historical control group limits control over temporal-related factors beyond those assessed herein. However, the consistency of the operators, all with extensive experience in all types of EVAR, limits the effects of a potential learning curve. While this may support internal validity, this study highlights the need for continuous auditing the results and improving the setup of the angiographic systems. In addition, the ultra-low-dose imaging protocol was implemented through system-specific parameter adjustments, which may limit direct reproducibility across institutions using different imaging platforms. In addition, renal outcomes, including the incidence and severity of acute kidney injury (AKI), were not systematically assessed in this study. However, no patients required renal replacement therapy during the study period. As group allocation followed an intention to treat principle, a brief transitional phase after protocol implementation meant that a small number of patients in the ultra-low-dose group were managed according to the previous imaging protocol, fully or partially. While this may have introduced minimal exposure to the older workflow within Group A, it is unlikely to have meaningfully influenced the overall results. The lack of statistical significance for DAP reduction in the small thoracic subgroup (*p* = 0.058) is likely a Type II error, given the clear 36% numerical reduction and the consistent pattern across all other subtypes. Future prospective, multi-center studies should validate these findings and assess the learning curve for protocol adoption. Long-term follow-up is required to ensure that ultra-low-dose imaging does not impact the durability of aneurysm exclusion.

## 5. Conclusions

An optimized, ultra-low-dose imaging workflow achieves substantial and consistent reductions in radiation and contrast exposure during EVAR without compromising technical success. By embedding dose minimization into the procedural environment, this integrated approach offers a scalable, system-level strategy to enhance safety for patients and procedural teams and should be considered a fundamental component of contemporary endovascular practice.

## Figures and Tables

**Table 1:** Baseline characteristics of the study cohort. Data are presented as median (interquartile range) or number (percentage). Group A (ultra-low-dose protocol, *n* = 228); Group B (standard low-dose protocol, *n* = 103). BMI, body mass index; IBD, iliac branch device; TEVAR, thoracic endovascular aortic repair.

Characteristic	Group A (*n* = 228)	Group B (*n* = 103)	*p*-Value
**Demographics**			
Age (years)	71 (66–76)	70 (66–75)	0.851
Male sex	193 (85%)	87 (85%)	0.966
BMI (kg/m^2^)	27 (24–30)	26 (24–30)	0.411
Comorbidities			
Hypertension	144 (63%)	80 (78%)	0.005
Baseline Creatinine (μmol/L)	92 (77–110)	93 (75–111)	0.977
**Procedure Type**			
Thoracoabdominal	22 (9%)	33 (32%)	<0.001
Juxtarenal	79 (33%)	21 (20%)	
Thoracic (TEVAR)	29 (12%)	10 (10%)	
Infrarenal	55 (23%)	22 (21%)	
IBD + Infrarenal	43 (18%)	17 (17%)	

**Table 2:** Intraoperative radiation dose and contrast. Use for the overall cohort. Values are median (interquartile range). DAP, dose-area product; DSA, digital subtraction angiography.

Metric	Group A (*n* = 228)	Group B (*n* = 103)	*p*-Value
Fluoroscopy time (s)	57 (29–86)	64 (33–101)	0.278
Fluoroscopy DAP (Gy·cm^2^)	26.0 (12.4–52.3)	47.5 (18.7–84.1)	0.001
DSA DAP (Gy·cm^2^)	27.2 (14.1–53.2)	129.9 (75.4–168.2)	<0.001
Total DAP (Gy·cm^2^)	57.9 (30.6–111.8)	199.3 (116.4– 302.8)	<0.001
Contrast volume (mL)	101 (61–139)	126 (78–171)	<0.001

**Table 3:** Procedure-specific radiation and contrast exposure. Total DAP and contrast volume are shown as median (IQR). Percentage reduction indicates median reduction in Group A compared to Group B. * A positive value denotes higher median contrast volume in Group A.

Procedure Type	Group	Total DAP (Gy·cm^2^)	DAP Reduction	Contrast (mL)	Contrast Reduction	Technical Success
Thoracoabdominal	A (*n* = 22)	109.9 (51.5–236.3)	58% (*p* < 0.001)	142 (98–167)	1% (*p* = 0.349)	17/22 (77%)
	B (*n* = 33)	262.9 (200.0–367.7)		143 (106–197)		28/33 (85%)
Juxtarenal	A (*n* = 79)	80.7 (41.4–128.2)	67% (*p* < 0.001)	108 (84–146)	14% (*p* = 0.528)	65/79 (82%)
	B (*n* = 21)	241.7 (140.4–432.0)		126 (84–166)		18/21 (86%)
Infrarenal	A (*n* = 55)	31.3 (19.4–58.4)	68% (*p* < 0.001)	74 (42–126)	12% (*p* = 0.368)	47/55 (85%)
	B (*n* = 22)	98.9 (83.4–164.7))		84 (49–136)		21/22 (95%)
IBD + Infrarenal	A *(n* = 43)	57.4 (28.8–93.8)	70% (*p* < 0.001)	85 (52–130)	36% (*p* = 0.005)	41/43 (95%)
	B (*n* = 17)	189.9 (130.9–234.9)		132 (85–185)		15/17 (88%)
Thoracic	A (*n* = 29)	58.1 (22.6–119.2)	36% (*p* = 0.058)	96 (61–139)	+81% * (*p* = 0.363)	27/29 (93%)
	B (*n* = 10)	90.4 (60.3–158.5)		53 (40–175)		8/10 (80%)

**Table 4:** **Impact of protocol adherence on radiation and contrast dose (Group A).** Patients in Group A were stratified by the proportion of DSA runs performed with the ultra-low-dose preset (<70% vs. ≥70%). Values are median (IQR).

Metric	<70% Low-Dose DSA (*n* = 69)	≥70% Low-Dose DSA (*n* = 149)	*p*-Value
Total DAP (Gy·cm^2^)	106.1 (52.4–192.9)	41.4 (25.5–81.7)	**<0.001**
Contrast volume (mL)	121 (82–146)	91 (56–126)	**0.002**
Fluoroscopy time (s)	56 (26–103)	58 (29–85)	0.785
Technical Success	58/69 (84%)	131/149 (88%)	0.435
BMI	27.4 (23.9–31.0)	26.9 (24.0–29.3)	*p* = 0.521

## Data Availability

Data are unavailable due to privacy.

## Abbreviations

The following abbreviations are used in this manuscript:

ALARA	As low as reasonably achievable
BMI	Body mass index
CBCT	Cone-beam CT
CO_2_	Carbon dioxide
DAP	Dose-area product
DSA	Digital subtraction angiography
EVAR	Endovascular aortic repair
IBD	Iliac branch device
IQR	Interquartile range
LOESS	Locally weighted scatterplot smoothing curve
TAAA	Thoracoabdominal aneurysm repair
TEVAR	Thoracic aneurysm repair

## References

[1] Greenhalgh R.M., Brown L.C., Kwong G.P., Powell J.T., Thompson S.G. (2004). Comparison of endovascular aneurysm repair with open repair in patients with abdominal aortic aneurysm (EVAR trial 1), 30-day operative mortality results: Randomised controlled trial. Lancet.

[2] Prinssen M., Verhoeven E.L., Buth J., Cuypers P.W., van Sambeek M.R., Balm R., Buskens E., Grobbee D.E., Blankensteijn J.D. (2004). A randomized trial comparing conventional and endovascular repair of abdominal aortic aneurysms. N. Engl. J. Med..

[3] Wanhainen A., Gombert A., Antoniou G.A., Fidalgo Domingos L.A., Gouveia E.M.R., Grabenwöger M., Haulon S., Katsargyris A., Kölbel T., Loftus I.M. (2026). European Society for Vascular Surgery (ESVS) 2026 Clinical Practice Guidelines on the Management of Descending Thoracic and Thoraco-Abdominal Aortic Diseases—Editor’s Choice. Eur. J. Vasc. Endovasc. Surg..

[4] Wanhainen A., Van Herzeele I., Bastos Goncalves F., Bellmunt Montoya S., Berard X., Boyle J.R., D’oRia M., Prendes C.F., Karkos C.D., Kazimierczak A. (2024). Editor’s Choice—European Society for Vascular Surgery (ESVS) 2024 Clinical Practice Guidelines on the Management of Abdominal Aorto-Iliac Artery Aneurysms. Eur. J. Vasc. Endovasc. Surg..

[5] Lima G.B.B., Dias-Neto M., Tenorio E.R., Baghbani-Oskouei A., Oderich G.S. (2022). Endovascular Repair of Complex Aortic Aneurysms. Adv. Surg..

[6] Modarai B., Haulon S., Ainsbury E., Böckler D., Vano-Carruana E., Dawson J., Farber M., Van Herzeele I., Hertault A., van Herwaarden J. (2023). Editor’s Choice—European Society for Vascular Surgery (ESVS) 2023 Clinical Practice Guidelines on Radiation Safety. Eur. J. Vasc. Endovasc. Surg..

[7] Hertault A., Maurel B., Midulla M., Bordier C., Desponds L., Saeed Kilani M., Sobocinski J., Haulon S. (2015). Editor’s Choice—Minimizing Radiation Exposure During Endovascular Procedures: Basic Knowledge, Literature Review, and Reporting Standards. Eur. J. Vasc. Endovasc. Surg..

[8] El-Sayed T., Patel A.S., Cho J.S., Kelly J.A., Ludwinski F.E., Saha P., Lyons O.T., Smith A., Modarai B., Tyrrell M. (2017). Radiation-Induced DNA Damage in Operators Performing Endovascular Aortic Repair. Circulation.

[9] Rajaraman P., Doody M.M., Yu C.L., Preston D.L., Miller J.S., Sigurdson A.J., Freedman D.M., Alexander B.H., Little M.P., Miller D.L. (2016). Cancer Risks in U.S. Radiologic Technologists Working With Fluoroscopically Guided Interventional Procedures, 1994–2008. AJR Am. J. Roentgenol..

[10] Roguin A., Goldstein J., Bar O., Goldstein J.A. (2013). Brain and neck tumors among physicians performing interventional procedures. Am. J. Cardiol..

[11] Davenport M.S., Perazella M.A., Yee J., Dillman J.R., Fine D., McDonald R.J., Rodby R.A., Wang C.L., Weinreb J.C. (2020). Use of Intravenous Iodinated Contrast Media in Patients With Kidney Disease: Consensus Statements from the American College of Radiology and the National Kidney Foundation. Kidney Med..

[12] Hertault A., Rhee R., Antoniou G.A., Adam D., Tonda H., Rousseau H., Bianchini A., Haulon S. (2018). Radiation Dose Reduction During EVAR: Results from a Prospective Multicentre Study (The REVAR Study). Eur. J. Vasc. Endovasc. Surg..

[13] McNally M.M., Scali S.T., Feezor R.J., Neal D., Huber T.S., Beck A.W. (2015). Three-dimensional fusion computed tomography decreases radiation exposure, procedure time, and contrast use during fenestrated endovascular aortic repair. J. Vasc. Surg..

[14] Edsfeldt A., Sonesson B., Rosén H., Petri M.H., Hongku K., Resch T., Dias N.V. (2020). Validation of a New Method for 2D Fusion Imaging Registration in a System Prepared Only for 3D. J. Endovasc. Ther..

[15] Hertault A., Bianchini A., Amiot S., Chenorhokian H., Laurent-Daniel F., Chakfé N., Lejay A. (2020). Editor’s Choice—Comprehensive Literature Review of Radiation Levels During Endovascular Aortic Repair in Cathlabs and Operating Theatres. Eur. J. Vasc. Endovasc. Surg..

[16] Hertault A., Maurel B., Sobocinski J., Martin Gonzalez T., Le Roux M., Azzaoui R., Midulla M., Haulon S. (2014). Impact of hybrid rooms with image fusion on radiation exposure during endovascular aortic repair. Eur. J. Vasc. Endovasc. Surg..

[17] Kaladji A., Dumenil A., Mahé G., Castro M., Cardon A., Lucas A., Haigron P. (2015). Safety and accuracy of endovascular aneurysm repair without pre-operative and intra-operative contrast agent. Eur. J. Vasc. Endovasc. Surg..

[18] Vaccarino R., Karelis A., Singh B., Marinko E., Tasopoulou K.M., Resch T., Sonesson B., Dias N.V. (2024). Assessment of Carbon Dioxide Angiography in Iliac Branched Repair. J. Endovasc. Ther..

[19] Serna Santos J., Uusi-Simola J., Kaasalainen T., Aho P., Venermo M. (2020). Radiation Doses to Staff in a Hybrid Operating Room: An Anthropomorphic Phantom Study with Active Electronic Dosimeters. Eur. J. Vasc. Endovasc. Surg..

[20] Finnesgard E.J., Simons J.P., Jones D.W., Judelson D.R., Aiello F.A., Boitano L.T., Sorensen C.M., Nguyen T.T., Schanzer A. (2023). Initial single-center experience using Fiber Optic RealShape guidance in complex endovascular aortic repair. J. Vasc. Surg..

[21] van Herwaarden J.A., Jansen M.M., Vonken E.P.A., Bloemert-Tuin T., Bullens R.W.M., de Borst G.J., Hazenberg C.E. (2021). First in Human Clinical Feasibility Study of Endovascular Navigation with Fiber Optic RealShape (FORS) Technology. Eur. J. Vasc. Endovasc. Surg..

[22] Hoell N.G., Erdely A., Goel V., Parodi F.E., Rowse J., Caputo F.J. (2026). Electromagnetic Intraoperative Positioning System (IOPS) is safe and effective as a 3D imaging adjunct in endovascular aortic surgery—A safety and feasibility study. Ann. Vasc. Surg..

